# Bio-Compounds, Antioxidant Activity, and Phenolic Content of Broccoli After Impregnation with Beetroot Juice

**DOI:** 10.3390/molecules30102143

**Published:** 2025-05-13

**Authors:** Magdalena Kręcisz, Joanna Kolniak-Ostek, Bogdan Stępień, Maciej Combrzyński

**Affiliations:** 1 Institute of Agricultural Engineering, Wrocław University of Environmental and Life Sciences, Chełmońskiego Street 37a, 51-630 Wrocław, Poland; bogdan.stepien@upwr.edu.pl; 2 Department of Fruit, Vegetable and Grain Technology, Wroclaw University of Environmental and Life Sciences, Chełmońskiego Street 37/41, 51-630 Wrocław, Poland; joanna.kolniak-ostek@upwr.edu.pl; 3 Department of Food Process Engineering, University of Life Sciences in Lublin, Głęboka 31, 20-612 Lublin, Poland; maciej.combrzynski@up.lublin.pl

**Keywords:** broccoli, beetroot, bioactive compounds, antioxidant activity, phenols, kinetics, vacuum impregnation, drying

## Abstract

The study presented in this paper examined the effects of vacuum impregnation (VI) with beetroot juice and drying medium temperature on selected properties of broccoli. Broccoli florets were dried using the convection-drying method (CD) at a constant drying factor speed (1 m/s) at temperatures of 50 and 70 °C. The bioactive compound content, antioxidant capacity, and polyphenol content of the broccoli before and after vacuum impregnation were determined. The Page and logistic models showed good compliance with the experimental data obtained for all of the tested versions of the materials. In addition, the water activity, density, and color were examined. The use of the vacuum impregnation process resulted in an increase in the drying time, the determination of six betalains characteristic of beetroots, an increase in the polyphenol content, and an increase in the antioxidant activity (FRAP). The material after VI was characterized by a darker color, a red color, and an increased density. Increasing the drying factor temperature resulted in a shorter drying time and a higher antioxidant activity value and content of polyphenols. These findings provide valuable insights into the relationship between VI, the drying temperature, and the broccoli tissue’s characteristics, offering guidance for optimizing processing conditions and the production of innovative dried materials that can be added to dishes or serve as a healthy snack.

## 1. Introduction

Broccoli (*Brassica oleracea* L. var *italica)* is one of the most widely grown vegetables in the world. It originates from the Mediterranean region and belongs to the cabbage family [1]. Since 2019, a doubling in broccoli production has been observed compared with the latter part of the 19th century. The largest producers of broccoli are India, the USA, Spain, and Italy. Broccoli is rich in bioactive compounds such as tocopherols, which are the most abundant in the Brassicaceae family. In addition, many glucosinolates have been identified in broccoli, which have been shown to prevent chronic diseases. Broccoli also contains carotenoids, such as lutein, β-carotene, neoxanthin, and violaxanthin [2]; minerals (zinc, selenium, iron, magnesium, potassium, calcium, and phosphorus); and ascorbic acid. The compounds listed above are probably responsible for reducing the risk of cancer and cardiovascular disease [3,4].

Due to its high betalain content, beetroot is among the top ten vegetables with the highest antioxidant properties. In addition, beetroot contains bioactive compounds, thanks to which its consumption has a positive effect on the human body [5].

Under normal temperature and humidity conditions (i.e., 25 °C and 50%), the quality of vegetables deteriorates rapidly. Fresh vegetables should be stored at temperatures below ambient temperature, i.e., around 15 °C. Otherwise, they must be packaged and frozen to enhance their storage stability [3]. Drying is an important step in the food industry that makes it possible to extend the shelf life of fruits and vegetables [6,7]. This is mainly due to the reduction in the water content in the product, which limits the growth of microbiological contaminants and prevents the occurrence of unwanted chemical reactions. Drying allows the preservation of nutrients, such as bioactive compounds (catechins, phenolic compounds, and isoflavones), protein, fiber, sugars, vitamins, and minerals [8,9,10]. An important advantage of dried materials is that they are easier and cheaper to transport due to their reduced volume and weight [11]. Other authors have shown that, of the various drying methods studied, samples obtained by the convection method have the highest-rated physicochemical properties, including bioactive ones, and thus, this method is optimal for drying quince [11] and coriander [12]. In the food industry, taking energy efficiency into account, more than 85% of all industrial thermal dryers are convectional [9]. Convective drying (CD) is still the most popular drying technique implemented in food-processing technologies due to the simplicity of the equipment, the financial efficiency of the method, and the preservation of bioactive compounds. Therefore, due to the numerous advantages of convective drying, it is crucial to use this technique to examine the content of bioactive components and optimize the drying process, especially since the modification of the plant material matrix affects the drying process [11,12,13].

The regular consumption of vegetables and fruits, which are a component of a balanced diet, has a positive effect on health, as proven by numerous studies [14]. In addition, increased consumer awareness and the extended promotion of a healthy lifestyle and nutrition have resulted in the significant growth of the functional food market and the search for effective methods of food enrichment. Vacuum impregnation (VI) is one of the methods that can be used before the drying process to enrich food, and it is also one of the non-destructive methods that allow the formation of a functional food with specific ingredients, such as probiotics, minerals, and vitamins, in order to improve its thermal, sensory, and bioactive properties [15,16]. This is possible thanks to the porous structure of vegetables and fruits. In the intercellular spaces of the materials, gas exchange with an aqueous solution takes place [17,18,19]. This occurs because the pressure in the cylinder is lowered, causing the pores to expand and the native gases to be displaced. The next stage of vacuum impregnation is called relaxation and is associated with the filling of the pores with the impregnating solution and their shrinkage [19]. VI is considered a valuable process that allows for the development of high-quality food products with desirable characteristics [18]. Pre-treatment can minimize unfavorable changes occurring in the tissue during drying and, as a result, obtain dried material of an appropriate quality [12]. VI with calcium ions can improve texture [20]. Other researchers have reported an increased firmness of honeydew melons after vacuum impregnation with calcium [21]. The use of grape juice resulted in an increase in bioactive compounds in apple tissue [22]. The type of impregnation solution has an impact on the qualitative and metabolic parameters of apples [17]. Vacuum impregnation has also been used to change the properties of beef. Researchers have observed that the use of onion juice reduces lipid oxidation and increases the taste rating of beef [23]. VI has also been used to enrich broad bean flour with iron. The results of these studies also confirmed the positive effect of vacuum impregnation [24]. In turn, De Olivier et al. [25] compared the impact of two pre-treatments on melon processing: soaking and vacuum impregnation. Beetroot juice was used in fruit and vegetable matrices and had a positive effect on the parameters studied [16,26]. The VI value largely depends on the structure of the fruit and vegetable tissue [15,27]. Popular impregnating solutions include calcium, iron, sucrose, probiotics, and sodium chloride. Currently, in addition to estimating the effect of VI on the physical properties of materials, the focus is on the bioactive properties of polyphenol content and antioxidant activity, which is why vegetable and fruit juices with high nutritional properties are used in the vacuum impregnation process, such as onion juice, kale juice, red beet juice, grape juice, or aloe vera gel [15,17,20,23,24,28]. Their research showed that the use of VI was a more effective method of maintaining the viability of probiotics. Vacuum impregnation as a pre-treatment method can be used to create new, innovative, and functional food. Current trends in the consumption of health-promoting foods indicate a greater demand in this sector. Therefore, it is necessary to use vacuum impregnation using beetroot juice with high bioactive properties to produce innovative dried products.

There is still a lack of comprehensive reports in the literature on the impact of convective drying and vacuum impregnation with beetroot juice on bioactive properties, antioxidant activity, polyphenols, drying kinetics, and selected physical properties, i.e., water activity, color, and density.

This review presents a comprehensive and innovative study of the latest trends in the creation of functional foods based on broccoli with beetroot juice. The aim of this study was to examine the effect of beet juice vacuum impregnation (VI) and drying agent temperature on selected bioactive and physical properties of fresh and dried broccoli and their potential industry applications in functional foods, emphasizing the novelty of the bioactive compounds in broccoli. The suitability of the vacuum impregnation process was evaluated through the analysis of the following parameters: bioactive compounds, antioxidant activity, polyphenols, drying kinetics, water activity, density, and color.

## 2. Results and Discussion

### 2.1. Bioactive Compounds of Broccoli Extracts

Table 1 shows the list of 30 phenolic compounds, eight glucosinolates, and six betalains identified in broccoli samples. Among the phenolic compounds, hydroxycinnamic acids, kaempferol, and quercetin derivatives were the dominant groups. After hydrolysis to aglycons of specific phenolic compounds, Fernandez-Leon et al. [29] investigated the bioactive components in fresh broccoli heads of the cultivars ‘Monaco’ and ‘Parthenon,’ finding a higher concentration of quercetin derivatives than kaempferol. According to Vallejo et al. [30], the predominant flavonoids in broccoli inflorescence samples after alkaline hydrolysis are complex acylated tri- or tetra-glycosides of kaempferol and quercetin. In the broccoli samples used in this study, 10 kaempferol glycosides and four quercetin derivatives were tentatively identified. Additionally, the analysis showed the presence of 15 derivatives of phenolic acids—mostly caffeic and sinapic acid derivatives.

Glucosinolates were identified in all tested samples (Table 2). We identified compounds typical for broccoli, whose presence in broccoli has been confirmed by other studies [31,32]. The compound at *m/z* 195 and the resulting product ions at *m/z* 177 and 129 were tentatively identified as gluconic acid [33]. The compound at *m/z* 436 and product ions at *m/z* 372 and 178 were identified as glucoraphanin [33], while the compound with the [M−H]^−^ ion at *m/z* 447 and product ions at *m/z* 259 and 134 was identified as glucobrassicin [33]. The compound at *m/z* 447 and resulting product ions at *m/z* 254 and 139 were identified as indolymethyl glucosinolate [33], while the compound at *m/z* 422 and product ions at *m/z* 358 and 259 were tentatively identified as glucoiberin [34]. Three compounds had the same [M−H]^−^ ions, but due to the different fragmentation patterns, they were identified as neoglucobrassicin (product ions at *m/z* 284 and 154) and two isomers of methoxyglucobrassicin (product ions at *m/z* 235).

In samples with the addition of beetroot juice, we identified six betalains, characteristic for beetroots (Table 3): 2,17-bidecarboxy-betanidin/isobetanidin ([M−H]^−^ ion at *m/z* 463 and resulting product ion at 301), vulgaxanthin ([M−H]^−^ ion at *m/z* 341), cyclodopa glucoside ([M−H]^−^ ion at *m/z* 356), N-formylcyclodopa glucoside ([M−H]^−^ ion at *m/z* 385), betanin ([M−H]^−^ ion at *m/z* 551 and resulting product ion at 389), and mono-decarboxy betanin ([M−H]^−^ ion at *m/z* 535) [5,35].

The process of vacuum impregnation is influenced by a variety of factors, which can be categorized into internal and external factors. Among variables of internal origin, features characteristic of impregnated tissue are distinguished, like its size, porosity, mechanical properties, and shape of capillaries. In turn, key external factors determining the impregnation rate encompass parameters such as the vacuum level applied, the concentration and composition of the impregnating solution, the proportion of the solution to the product, the temperature, the intensity of mixing, the vacuum process duration, and the moment of the restoration of atmospheric pressure [28,36].

The use of vacuum impregnation technology not only allowed the introduction of new compounds characteristic of beet, but also a significant increase in other compounds also present in fresh broccoli. The largest change was noted for quercetin-3,4′-*O*-di-beta-glucoside; the use of VI allowed the tested compound to be increased by 20,464%. Significant changes in the content of bioactive compounds after the vacuum impregnation process were also noted for trisinapoyl-gentionbiose (3,056%), caffeoylquinic acid (1 381%), 1,2-diferuoyl-diglucoside (738%), orientin (723%), coumaroyl-quinic acid (396%), 1,2-disinapoyl-gentobioside (328%), kempferol-3-sinapoylsophorotrioside-7-glucoside (265%), caffeoyl hexoside (235%), kempferol-3-caffeoyltriglucoside-7-glucoside (200%), and feruloyl-disinapoyl-gentionbiose (194%). However, in these studies, the total content of bioactive compounds decreased after the vacuum impregnation process. This indicates the characteristics of the vacuum impregnation process and the mass transfer between the vegetable tissue and the impregnation solution [36]. The vacuum impregnation process can increase the nutritional value of products, but at the same time, changes in the structure can lead to the degradation of some bioactive compounds due to contact with oxygen or enzymes [36]. Studies by other authors have shown that appropriate VI parameters (i.e., time and pressure) can increase the content of phenols and flavonoids in apples. However, excessive process intensity can cause losses of these compounds [37].

### 2.2. Total Phenolic Content (TPC) and Antioxidant Capacity (AC)

The results for antioxidant activity and fresh polyphenols (B), after vacuum impregnation with beetroot juice (BB) and dried broccoli florets at 50 °C (CD50) and 70 °C (CD70), are shown in Table 4.

Antioxidant capacity (AC) was tested using the ABTS, DPPH, and FRAP methods. The highest AC value for ABTS and DPPH was recorded for fresh broccoli (88.60 µMol/g), and the lowest was recorded for broccoli with VI after drying at 50 °C (16.57 µMol/g). Other authors, studying the effect of VI with the use of apple and pear juice on the properties of chokeberry, observed a decrease in antioxidant activity. In addition, the authors observed that with the increase in pressure during the vacuum impregnation process, the AC value decreases [38]. The impregnation process is influenced by many agents, such as material structure, vacuum pressure, VI time, impregnating solution, and solution particle size [28]. In the case of the FRAP method, the highest value was recorded for broccoli after the vacuum impregnation process (47.79 µMol/g), and the lowest was recorded for broccoli without VI after drying at 50 °C (13.20 µMol/g). The samples dried after VI had a lower AC than the samples dried without pre-treatment. Matys. et al., who studied the effect of pre-treatment with pulsed electric fields in their studies, obtained observations consistent with those presented in this study [13]. Other studies show that in the case of drying sweet potatoes, a higher AC value was recorded when a vacuum impregnation process was used with onion juice and kale [39] and for celery after the VI of onion juice, kale, and celery leaves [40]. A higher antioxidant activity value, tested by ABTS, DPPH, and FRAP methods in broccoli dried by convection at 70 °C, was observed by 54.96, 2.80, and 73.64% for broccoli without VI and by 41.84, 46.89, and 27.23% for broccoli after VI compared to broccoli dried at a lower temperature. Higher AC values with increasing drying process temperatures were observed by other researchers for spray-dried broccoli stem and broccoli florets powders [3].

The used VI process resulted in the highest polyphenol value (13.06 µMol/g). In the case of broccoli not subjected to the drying process, the TPC value was 32.99% higher in broccoli after VI (BB) than for broccoli without pre-treatment. In the study, it was observed that the temperature of the drying medium influenced the increase in polyphenol value by 62.5% in the case of untreated broccoli and by 33.08% for broccoli after impregnation with beetroot juice. An increase in TPC with increasing temperature from 50 to 70 °C during convective drying was observed in other studies in which celery and celery after VI were dried using onion juice, kale, and celery [40]. Saavedra-Leos et al. [3] observed that the TPC value increased with increasing temperature from 170 to 220 °C when spray-drying broccoli stems and florets [3]. Similar observations were made by Sarabandi et al. [41] when spray-drying eggplant peel extracts enclosed in gum arabic and maltodextrin. The authors explained that the increase in TPC at higher temperatures may be due to the faster formation of a protective film around the materials studied. As a result, more polyphenols are enclosed in the microstructure of the materials [41]. In addition, a greater loss of polyphenols was observed in the material dried after VI by 11.15% after CD50 and by 27.23% after CD70. These losses may be related to the partial loosening of the broccoli tissue. This loosening can lead to the leakage of the tested solutions and the activation of reaction substrates and/or oxidizing enzymes (flavonoids and phenols) from the vegetable materials [13,42]. Other studies indicate an increase in TPC value for sweet potatoes impregnated with onion juice and kale [39]. Many researchers report that the changes in the phenolic compound values can be influenced by agents such as the type of raw material, process temperature, pre-treatment time, and the type of osmotic compound [14,43,44].

### 2.3. Vacuum Impregnation

The weight gain (WG), Brix degrees (°Bx), and dry mass (DM) are shown in Table 5. In this study, in which broccoli juice was used, the weight gain of the broccoli was 14.88% at 10.3 °Bx, compared to the broccoli before vacuum impregnation. This indicates the porous structure of the tested material. Other authors, studying the effect of the vacuum impregnation process with the use of an isotonic solution (13–14 °Bx) on the properties of apples, observed a WG of 20.1–23.4% [17].

### 2.4. Moisture Ratio Curves and Water Content Curves

**Figure 1:**
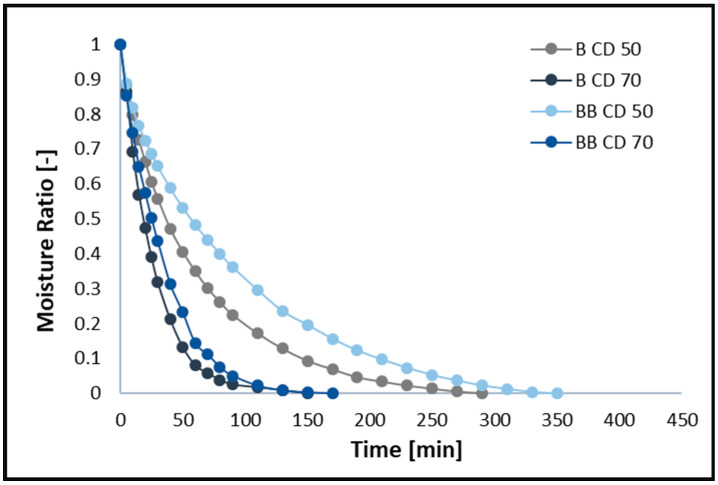
Drying kinetics for convective drying at 50 °C (CD50) and 70 °C (CD70). B—broccoli without VI; BB—broccoli after VI.

**Figure 2:**
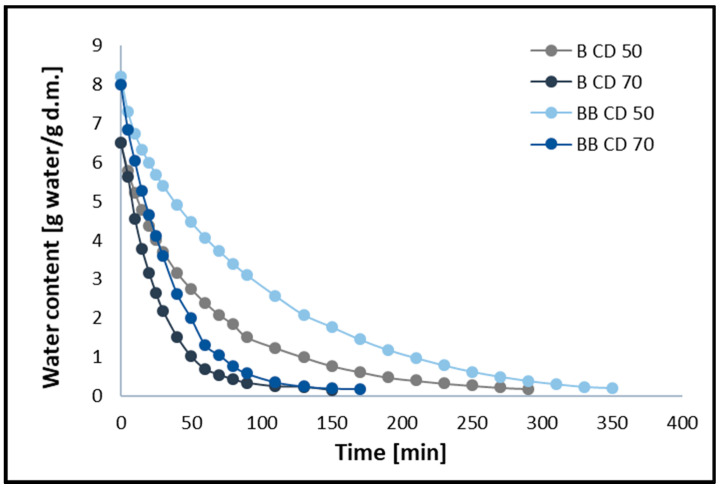
Water content during convective drying at 50 °C (CD50) and 70 °C (CD70). B—broccoli without VI; BB—broccoli after VI.

The changes in moisture ratio (MR) and moisture content (MC) of broccoli samples without pre-treatment (B) and after vacuum impregnation (BB) dried using the CD method at 50 and 70 °C are shown in Figure 1 and Figure 2, respectively. The figures show that different drying temperatures and the use of vacuum impregnation (VI) had a significant impact on the change in moisture content and drying time in all samples tested. The moisture content of broccoli florets decreases with increasing temperature, and thus the drying time decreases. The time required to reach the equilibrium moisture content of the broccoli was 150 (B CD70), 170 (BB CD70), 290 (B CD50), and 350 (BB CD50) minutes. The required drying time for CD 70 was 48.28% shorter for broccoli without pre-treatment and 51.43% shorter for broccoli after the VI process compared to convective drying at 50 °C. These studies confirm that increasing the temperature of the drying medium improves the diffusion and evaporation of moisture, thus shortening the drying time. These results are in line with previous studies on broccoli stems [45], carrots [46], coriander [12], celery [40,47], and green bananas [6].

The use of the vacuum impregnation process, using beet juice as an impregnating solution, resulted in an increase in drying time. In broccoli samples dried at 50 °C (CD50) with VI, the drying time increased by 20.69%, and in samples dried at 70 °C (CD70), it increased by 20%. In the material after the vacuum impregnation process, an increase in water content of 13.33% was observed, which probably contributes to the prolonged drying time of broccoli (BB). In addition, the weight gain in the VI samples was 14.88%, which may also contribute to the prolonged drying time.

### 2.5. Modeling of Broccoli Drying Process

The model parameters and statistical parameters describing the kinetics of the broccoli drying process are presented in Table 6. For convective drying at 50 and 70 °C, all five models provided an excellent fit to the experimental data with an R^2^ value above 0.99. Of these, the logistic model proved to be the best model for convective drying at 70 °C, taking into account the highest R^2^ values (0.9995 and 0.9992) and the lowest RSME values (0.0072 and 0.0093), V_e_ (2.1 and 2.9), and χ^2^ (0.0001). Interestingly, in the case of convective drying at 50 °C, the Page model achieved the best fit in terms of high R^2^ values (0.9995 and 0.9961) and the lowest RSME values (0.0075 and 0.0203). The best fit of the logistic model was demonstrated by researchers drying celery [47]. Other researchers have shown the best fit of Page’s model when drying quince [11], and this model is also commonly used in other studies on drying coriander [12].

### 2.6. Dry Matter (DM), Water Activity (AW), and Density (ρb)

The water activity (AW) for all tested material versions is shown in Table 7. As expected, the highest values of the tested parameter were recorded for fresh broccoli (0.987) and broccoli after the vacuum impregnation process (0.984). This is consistent with previous studies [26]. Anaya-Esparza et al., while studying the effect of vacuum impregnation on the properties of apples, observed an increase in water activity in apples after VI [37]. The use of the drying process allowed for a reduction in the AW by 64.01–52.45%, depending on the drying process temperature and the pre-treatment used. Lower water activity values were recorded for broccoli without VI: 0.469 (B CD50) and 0.436 (B CD70). Increasing the drying factor temperature resulted in a 18.49% reduction in water activity for fresh broccoli without VI and a 18.72% reduction for broccoli after VI. This is in line with our previous studies on broccoli [26], courgette [26,48], and celery [40].

The density for fresh broccoli was 240.94 kg/m^3^, and that for broccoli after the vacuum impregnation process was 288.90 kg/m^3^. As can be seen from the data presented in Table 5, broccoli after VI before and after drying are characterized by a higher density. These results are consistent with previous studies by the authors of the publication, which concerned celery impregnated with beetroot juice [47] and courgette impregnated with tomato juice [49]. The use of a higher drying factor temperature resulted in a lower density of the tested material. A decrease in the density of the tested material with increasing temperature was observed in previous studies when drying celery [47] and courgette [49].

### 2.7. Color

In the study presented in this paper, the color parameters (L*, a*, b*) of fresh broccoli, after the vacuum impregnation process, and dried broccoli were determined. Then, the saturation, browning, and color difference in relation to fresh material without VI were calculated, and the results are presented in Table 8.

The vacuum impregnation process reduced the lightness (L*) and the b* value, which indicates a yellow tint. In addition, the a* value was higher and took on positive values, indicating a red tint. These results are in line with expectations because beet juice, which has an intense dark color, was used in the vacuum impregnation process. These results are consistent with studies that examined courgette and broccoli [39], celery [38], and apples [37].

The use of a higher drying factor temperature resulted in a decrease in the L* and b* parameters and an increase in the a* parameter. The higher a* values can be explained by the active enzymatic and non-enzymatic browning (Maillard reaction) during drying, which led to a red-brown color of the samples both with and without VI [50,51,52]. On the other hand, the darker color of broccoli may be caused by the high temperature of the process [53]. Szychowski et al. [11], when drying quince, also observed the darkest color in samples dried at 70 °C [11].

The smallest change in color was observed for broccoli after VI without drying. The application of the drying process in broccoli after VI resulted in an increase in ∆E of 50.42% at CD50 and an increase of 68.22% at CD70. As predicted, a higher ∆E value was observed in the material after the vacuum impregnation process. This is consistent with the darker color of the samples (L*). In other studies, which examined the effect of tomato juice on the properties of courgette [49], kale and onion juice on the properties of courgette [48], and beet juice on the properties of celery [47], a similar relationship was observed. In addition, the greater color difference is due to the use of a strongly colored juice as an impregnation solution. The change in color difference is also confirmed by the browning index (BI). Higher values of the index were recorded in the material after the vacuum impregnation process. However, the highest change in this index was recorded in the samples after the drying process. The color saturation (C*) was recorded for BB and was the highest for B CD50. A decrease in the value of the tested parameter was observed in the material after VI. The drying process caused an increase in saturation, but as the temperature of the drying agent increased, the saturation value decreased.

### 2.8. Pearson Correlation of Drying Kinetics and Selected Broccoli Quality Attributes

**Figure 3:**
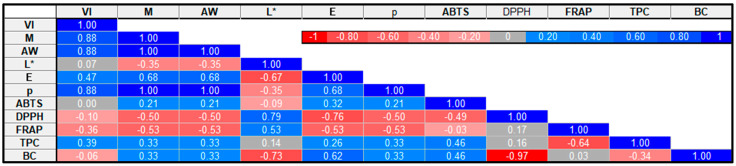
Correlation matrix for selected broccoli parameters. VI: vacuum impregnation; M: material with or without drying; AW: water activity; L*: lightness; E: color difference; p: bulk density; ABTS, DPPH, FRAP: antioxidant capacity; TPC: total phenolic content; and BC: bioactive compounds.

As shown in Figure 3, Pearson’s correlation was used to examine the association between selected broccoli properties. The data showed a significant (*p* < 0.05) positive correlation between vacuum impregnation and the material with or without drying (r = 0.88), water activity (r = 0.88), and bulk density (r = 0.88). The results also showed a significant positive correlation of the material with or without drying with water activity (r = 1), color difference (r = 0.68), and bulk density (r = 1) and a negative correlation with FRAP (r = −0.53) and DPPH (r = −0.50). The data obtained indicate that the material with or without drying had a greater impact on BC, FRAP, and ABTS than the drying methods. Both variables had a similar effect on water activity, color difference, bulk density, and total phenolic content. Moreover, a significant positive correlation was observed between water activity and bulk density (r = 1) and color difference (r = 0.68). Water activity negatively correlates with FRAP (r = −0.53) and ABTS (r = −0.50). The results also showed a positive correlation of ABTS with TPC and BC (r = 0.46) and a negative correlation with DPPH (r = −0.49). DPPH significantly negatively correlates with bioactive compounds (r = −0.93). The negative correlation between the content of bioactive compounds and the DPPH value is beneficial because it indicates the high ability of these compounds to neutralize free radicals, which indicates their strong antioxidant properties. Such action may contribute to protecting the body from oxidative stress and related diseases.

### 2.9. Principal Component Analysis (PCA)

**Figure 4:**
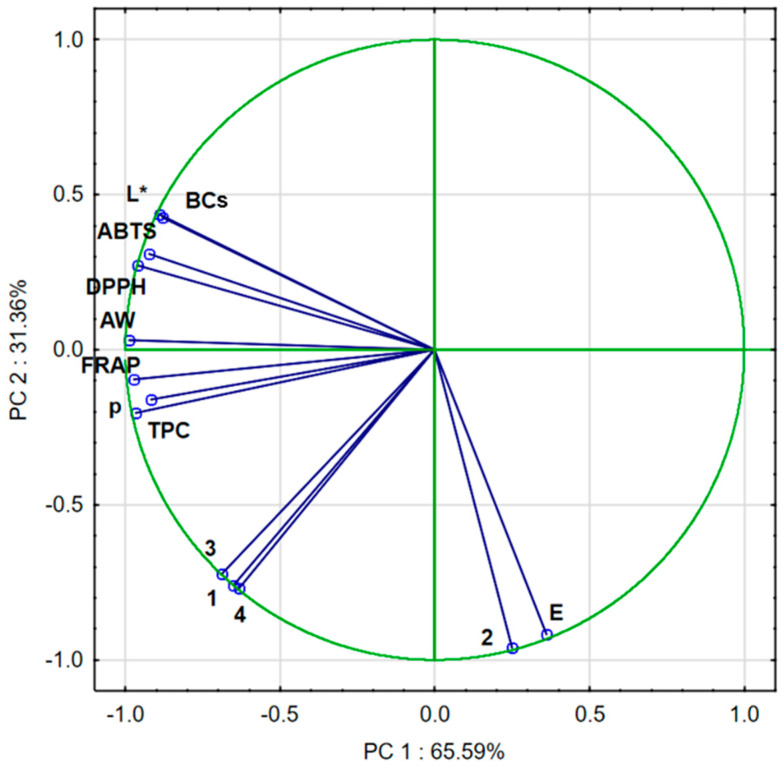
PCA of dried broccoli. AW: water activity; L*: lightness; E: color difference; p: bulk density; ABTS, DPPH, and FRAP: antioxidant capacity; TPC: total phenolic content; BCs: bioactive compounds; 1—quercetin-3,4′-*O*-di-beta-glucoside; 2—trisinapoyl-gentionbiose; 3—1,2-diferuoyl-diglucoside; and 4—caffeoylquinic acid.

Approximately 96.95% of the variance in the sample data was explained by the first two principal components PC1 (65.59%) and PC2 (31.36%). PC1 correlated negatively with DPPH, ABTS, FRAP, L*, BC, AW, p, TPC, quercetin-3,4′-O-di-beta-glucoside, 1,2-diferuoyl-diglucoside, and caffeoylquinic acid and positively with ∆E and trisinapoyl-gentionbiose. PC2 correlated negatively with BC, DPPH, ABTS, L*, TPC, and AW and positively with FRAP, p, TPC, quercetin-3,4′-*O*-di-beta-glucoside, trisinapoyl-gentionbiose, 1,2-diferuoyl-diglucoside, caffeoylquinic acid, and ∆E (Figure 4).

**Figure 5:**
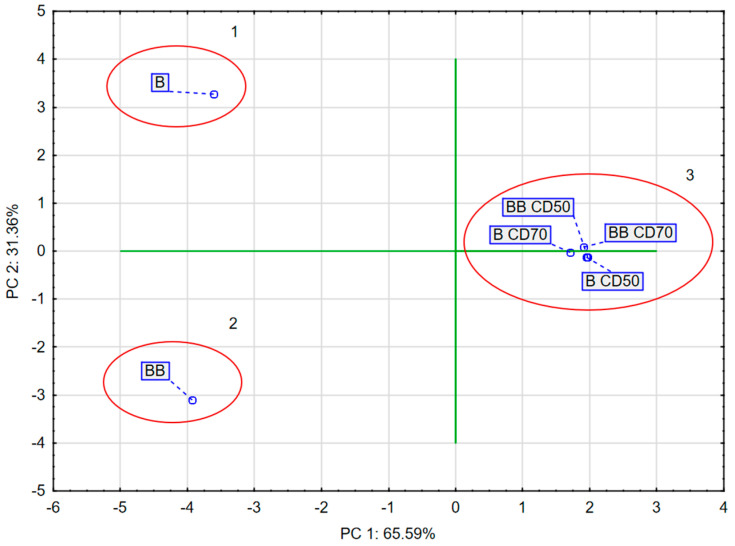
PCA of dried broccoli.

The PCA diagram (Figure 5) reveals that the first component (PC1) describes the results of broccoli after the drying process by 65.59%. In the figure, positive PC1 values describe the results obtained for broccoli after the drying process. Negative PC1 values describe fresh broccoli, i.e., not subjected to the convective drying process. The second main component (PC2) refers to the pre-treatment used, which was vacuum impregnation, at 31.36%. In the figure, positive PC2 values describe fresh broccoli after vacuum impregnation. Negative PC2 values describe the results of fresh broccoli and broccoli after convective drying. In addition, three groups were observed which indicated significant differences. The first group included fresh broccoli, the second group included broccoli after vacuum impregnation, and the third group included samples of broccoli after convective drying.

## 3. Materials and Methods

### 3.1. Plants

This study was conducted using broccoli purchased at a local vegetable market. The vegetables were stored in a refrigerator at 4 ± 1 °C (Samsung Electronics, Wronki, Poland). Before processing, the broccoli was washed, dried, and cut into evenly sized florets (1.5 cm in diameter and 2 cm in height).

### 3.2. Impregnation Solution

In the conducted research, organic, cold-pressed red beet juice was used as an impregnation solution (Haus Rabenhorst, Unkel, Germany). The average values per 100 mL were as follows: energy: 155 kJ (37 kcal), fat: <0.5, carbohydrate: 8.0 g, fiber: <0.5 g, protein: <0.5 g, and salt: 0.13 g.

### 3.3. Pre-Treatment Before Drying Process

A vacuum impregnation (VI) process was used as the pre-treatment. VI was carried out according to the procedure described in our previous publication [26]. For each pre-treatment, three independent vacuum impregnations were performed. Weight gain (WG) was calculated according to the method by Tappi et al. (2022) [17]:

(1) $$WG=100\cdot \frac{m-m_{0}}{m_{0}}$$

where

m—mass of the impregnated sample;
m_0_—initial mass.

After the vacuum impregnation process, the samples (Table 9) were analyzed for selected bioactive properties and physical properties, and the remaining samples were subjected to a drying process.

### 3.4. Convective Drying (CD)

The convective drying process was carried out at two set temperatures, 50 °C (CD50) and 70 °C (CD70), and an air flow of 1 m·s^−1^. Broccoli weights of 80 g were placed in six baskets, and the entire process was carried out in a drought in a dryer designed and built at the Institute of Agricultural Engineering (Wrocław, Poland) [54].

### 3.5. Bioactive Compounds, Antioxidant Capacity, and Phenols

#### 3.5.1. Extraction Procedure

The dried samples were ground in a laboratory mill (IKA 11A, Staufen, Germany) and then sent for extraction. An amount of 0.5 g of powder samples was extracted with 2 mL of 80% methanol acidified with 1% HCl (*v*/*v*) for 24 h under refrigeration. The samples were subjected twice to sonication (300 W, 40 kHz; Sonic 6D, Polsonic, Warsaw, Poland) for 20 min with periodic shaking. The slurry was centrifuged at 19,000× *g* for 10 min. The supernatant was then filtered over a hydrophilic PTFE 0.20 μm membrane (Millex SamplicityTM filter, Merck, Darmstadt, Germany) and used for analysis.

#### 3.5.2. Identification of Bioactive Compounds by UPLC-PDA–MS Method

The bioactive compounds in the broccoli samples were identified using an ACQUITY Ultra-Performance LC system (Waters Corporation, Milford, MA, USA) equipped with a photodiode array detector with a binary solvent manager and a mass detector G2 Q-TOF micro-mass spectrometer (Waters, Manchester, UK) equipped with an electrospray ionization (ESI) source operating in negative mode. Individual compound separations were performed at 30 °C using a UPLC BEH C18 column (1.7 µm, 2.1 mm, 100 mm, Waters Corporation, Milford, MA, USA). The samples (10 µL) were injected, and the elution took 15 min using the linear gradients and isocratic flow rates described previously by Kolniak-Ostek and Oszmiański [55]. Full-scan, data-dependent MS scanning from *m/z* 100 to 2500 was used for the analysis. The retention duration and precise molecular masses were used to characterize the individual components. Before and after fragmentation, each chemical was optimized to its predicted molecular mass in the negative mode. The retention periods and spectra were compared to genuine standards.

#### 3.5.3. Antioxidant Capacity (AC) and Total Phenolic Content (TPC)

In these studies, three methods of antioxidant capacity were used: DPPH [56], ABTS [57], and FRAP [58]. AC was determined using a Synergy H1 plate reader (BioTek, Winooski, VT, USA). Various concentrations of Trolox were used to prepare standard curves. All data obtained are expressed as Trolox equivalents per 100 g of broccoli dry matter (mMol Tx/100 g DM).

TPC was determined by using a modified method previously presented by Gao et al. [59]. In this study, we used 5 µL of methanolic broccoli extract, 10 μL of Folin–Ciocalteu reagent, 100 μL of H_2_O, and 50 μL of 10% sodium carbonate, and then everything was mixed and shaken for 30 s. Total phenolic content was read after 1 h. The measurements were recorded at 765 nm on a Synergy H1 (BioTek, Winooski, VT, USA) and saved as mg of gallic acid per 1 g of broccoli dry matter (DM).

### 3.6. Mathematical Modeling

The moisture ratio (MR) was determined using the following equation [60]:

(2) $$MR=\frac{M_{t}-M_{e}}{M_{0}-M_{e}}$$

where M_t_, M_e_, and M_0_, denote the moisture content achieved after the drying time, the equilibrium moisture content, and the initial moisture content, respectively.

Table 10 presents five popular mathematical models that were used in this study. A comparison of these models provided an opportunity to select the best model that describes convective drying.

### 3.7. Physical Properties

#### 3.7.1. Dry Weight

The dry weight of broccoli was determined using the vacuum dryer (Memmert, VO101, Schwabach, Germany) at 70 °C for 24 h under reduced pressure (5 kPa). The result was recorded as the average of three repetitions.

#### 3.7.2. Water Activity

Water activity was measured at constant temperature (25 °C) using AquaLab 4TE ± 0.003 (AquaLab, Warsaw, Poland). Four measurements were taken and the final result was the average.

#### 3.7.3. Bulk Density

The bulk density measurement was carried out using a measuring cylinder into which the tested material was poured, ensuring its even distribution without excessive compaction [65]. To increase the reliability of the results, the measurement was repeated six times. The bulk density was calculated based on the obtained results using the following formula:

(3) $$\rho_{b}=\frac{w_{s}}{V}$$

where ρ_b_, w_s_, and V denote the bulk density [kg·m^−3^], the weight of the samples [kg], and the volume [m^3^], respectively.

#### 3.7.4. Color

In order to examine the influence of the vacuum impregnation process, using beet juice as an impregnation solution and the temperature of the drying agent, three parameters were measured: L*, a*, and b*. The color of the broccoli was measured using a Minolta Chroma Meter CR-200 (Minolta Corp., Osaka, Japan). The color meter was calibrated against a standard white calibration plate. The values L*, a*, and b* were the average of 10 readings. The lightness coordinate L* measures the whiteness of the color and ranges from white at 100 to black at zero. The chromaticity coordinate a* determines the redness when the values are positive and the greenness when the values are negative. The third color coordinate b* measures the shade of yellow when the values are positive and the color blue when the values are negative. In addition, the following are calculated based on the three chromaticity coordinates: chroma (C*), browning index (BI), and color difference (ΔE) [47].

### 3.8. Statistical Analysis

Statistical analyses were performed using Statistica version 13.1 (StatSoft, Tulsa, OK, USA). A one-way analysis of variance (ANOVA) was used to assess differences between mean values, supplemented with Duncan’s test as a post hoc method. Differences were considered statistically significant at the significance level of *p* < 0.05. Additionally, principal component analysis (PCA) was performed to determine the relationships between physicochemical properties of broccoli and the drying method and vacuum impregnation process.

## 4. Conclusions

In this study, a vacuum impregnation process using beetroot juice and different hot-air drying temperatures (50 and 70 °C) was demonstrated to influence both the physical properties and bioactive compounds, antioxidant capacity, and polyphenol content of broccoli.

The best fit of the models to the experimental data was obtained using the modified Page model and a logistic model. In general, the process that gave good results and is the recommended drying method was CD at 70 °C; it showed higher values of bioactive compounds, antioxidant capacity, and polyphenols. The drying time required for CD 70 was 48.28% shorter for broccoli without pre-treatment and 51.43% shorter for broccoli after the VI process compared to convective drying at 50 °C.

The use of the vacuum impregnation process resulted in a 20.69% increase in drying time for broccoli samples dried at 50 °C (CD50) with VI and a 20% increase for samples dried at 70 °C (CD70). In the samples after VI, a higher polyphenol content and antioxidant capacity measured by the FRAP method were recorded. Additionally, bioactive compounds characteristic of beetroot were identified. In addition, the use of VI increased the density of the tested materials. The use of VI lowered the brightness value, which is related to the drying process and the type of impregnating solution used, and a higher red value was recorded.

In conclusion, the findings of this study provide crucial insights for industrial applications, suggesting that the vacuum impregnation process using beetroot juice may change the physical and chemical properties of broccoli. This research can provide inspiration for the development of innovative, healthy, and functional vegetable snacks. The vacuum impregnation process and the type of impregnation solution used significantly affect the properties of the vegetables studied. In addition, drying temperature is a core parameter for broccoli processing, which affects its properties and processing time. Future research may focus on the use of equally bioactive impregnation solutions and the application of additional drying methods such as microwave drying or vacuum drying. Therefore, the findings in this study serve as a platform for future research on the production of dried vegetables and the high-value and innovative usage of broccoli.

## 5. Patents

Patent Poland, no. 421913. Vacuum impregnating machine and method for initial processing of material. Wrocław University of Environmental and Life Sciences, Wrocław, PL. Authors: Bogdan Stępień, Radosław Maślankowski, Leszek Rydzak, and Marta Pasławska.

## Figures and Tables

**Table 1:** Bioactive compounds of broccoli (B) and broccoli impregnated in beetroot juice (BB) dried by convective drying at 50 °C (CD50) and 70 °C.

Rt (min)	[M−H]^−^ (*m/z*) ^a^	MS/MS Fragments (*m/z*) ^a^	Compound	B	B CD50	B CD70	BB	BB CD50	BB CD70
2.56	341.0826	179.0250/161.0179	Caffeoyl hexoside	16.02 ± 0.25 ^e^	0.25 ± 0.04 ^b^	0.40 ± 0.12 ^c^	4.60 ± 0.62 ^d^	0.19 ± 0.05 ^a^	0.19 ± 0.06 ^a^
3.28	353.0844	191.0560/179.0348	Caffeoylquinic acid	87.02 ± 2.15 ^f^	38.34 ± 1.15 ^e^	33.39 ± 1.18 ^d^	23.89 ± 1.26 ^c^	13.57 ± 0.74 ^a^	15.91 ± 0.84 ^b^
3.35	353.0811	179.0290	Caffeoylquinic acid	0.49 ± 0.04 ^b^	0.29 ± 0.11 ^a^	0.66 ± 0.09 ^c^	7.26 ± 0.35 ^e^	0.94 ± 0.04 ^d^	0.92 ± 0.14 ^d^
3.40	447.0410	357.0850	Orientin ^b^	0.80 ± 0.02 ^c^	0.56 ± 0.54 ^b^	0.28 ± 0.03 ^a^	7.01 ± 0.24 ^d^	0.20 ± 0.26 ^a^	0.19 ± 0.03 ^a^
3.85	609.1469	447.0531/285.0361	Kaempferol diglucoside	239.69 ± 4.13 ^e^	4.85 ± 0.16 ^a^	5.17 ± 0.12 ^b^	41.04 ± 1.32 ^d^	8.28 ± 0.86 ^c^	4.61 ± 0.24 ^a^
4.03	337.0337	191.0497/163.0337	Coumaroyl-quinic acid	1.94 ± 0.06 ^c^	1.18 ± 0.04 ^b^	1.27 ± 0.15 ^b^	9.61 ± 0.36 ^d^	0.79 ± 0.26 ^a^	0.89 ± 0.36 ^a^
4.20	353.0904	191.0596/	Caffeoylquinic acid ^b^	21.95 ± 1.18	1.25 ± 0.14	1.95 ± 0.08	16.35 ± 1.65	1.78 ± 0.42	1.44 ± 0.05
4.22	1111.3350	949.2182/301.0816	Quercetin-3-caffeoyltriglucoside-7-glucoside	2.69 ± 0.08 ^c^	1.11 ± 0.06 ^b^	0.27 ± 0.06 ^a^	5.50 ± 0.54 ^d^	0.41 ± 0.32 ^a^	5.41 ± 0.23 ^d^
4.48	1095.0083	933.4816/787.2160	Kempferol-3-caffeoyltriglucoside-7-glucoside	6.31 ± 0.04 ^c^	12.09 ± 0.45 ^d^	11.24 ± 0.12 ^d^	1.33 ± 0.26 ^a^	5.69 ± 0.16 ^b^	13.77 ± 0.54 ^e^
4.83	1139.2931	977.2447/285.0431	Kempferol-3-sinapoylsophorotrioside-7-glucosided	2.03 ± 0.19 ^a^	2.28 ± 0.08 ^a^	2.64 ± 0.20 ^b^	3.84 ± 0.36 ^c^	4.39 ± 0.54 ^d^	3.00 ± 0.64 ^c^
4.94	385.1083	205.0451/189.0714	Sinapoyl hexose	56.49 ± 2.53 ^g^	7.63 ± 0.34 ^d^	4.10 ± 0.26 ^b^	10.60 ± 0.64 ^f^	5.99 ± 0.12 ^c^	1.21 ± 0.08 ^a^
5.80	609.1398	447.0898/285.0439	Kaempferol diglucoside	68.74 ± 2.64 ^d^	11.12 ± 0.04 ^a^	10.04 ± 0.42 ^a^	31.68 ± 0.64 ^c^	11.14 ± 0.24 ^a^	18.58 ± 01.24 ^b^
6.51	341.0989			12.58 ± 1.24 ^b^	18.13 ± 1.06 ^c^	12.20 ± 0.65 ^b^	31.01 ± 0.68	10.38 ± 0.46 ^a^	18.10 ± 0.67 ^c^
6.53	301.0023	723.1643/429.1104	Quercetin ^b^	11.52 ± 1.36 ^a^	38.35 ± 1.74 ^c^	19.17 ± 0.34 ^b^	60.20 ± 1.12 ^d^	17.53 ± 0.84 ^b^	25.00 ± 1.29 ^b^
6.91	359.1560	197.0451/179.1125/161.0216	Rosmarinic acid ^b^	1.62 ± 0.02 ^b^	2.32 ± 0.06 ^c^	2.23 ± 0.08 ^c^	0.90 ± 0.04 ^a^	2.78 ± 0.22 ^d^	4.23 ± 0.46 ^e^
7.15	947.2666	785.2501/293.0054	Kempferol-3-feruloyldiglucoside-7-glucoside	47.88 ± 2.54 ^e^	8.61 ± 0.13 ^c^	6.97 ± 0.64 ^b^	111.59 ± 2.65 ^f^	5.28 ± 0.46 ^a^	23.65 ± 0.58 ^d^
7.35	1095.2855	787.1799/285.1093	Kempferol-3-caffeoyltriglucoside-7-glucoside	5.90 ± 0.42 ^a^	24.87 ± 0.54 ^e^	15.52 ± 0.16 ^b^	17.72 ± 1.12 ^c^	22.98 ± 0.98 ^d^	17.24 ± 1.16 ^c^
7.58	577.1445	285.0321	Kempferol-3,7-*O*-di-rhamnopyranoside	15.28 ±0.65 ^f^	5.26 ± 0.32 ^d^	1.06 ± 0.08 ^a^	10.56 ± 1.24 ^e^	1.81 ± 0.21 ^c^	1.42 ± 0.28 ^b^
8.12	919.3005	723.1643/429.1104/301.0721	Quercetin derivative	10.12 ± 0.24 ^c^	1.62 ± 0.14 ^a^	1.90 ± 0.16 ^b^	9.28 ± 1.16 ^c^	2.13 ± 0.08 ^b^	2.02 ± 0.62 ^b^
8.31	753.2167	523.1585/223.0616/205.0515	1,2-Disinapoyl-gentobioside	5.40 ± 0.16 ^a^	70.13 ± 2.25 ^d^	74.18 ± 2.14 ^d^	23.12 ± 1.54 ^b^	90.89 ± 2.54 ^e^	48.47 ± 2.12 ^c^
8.60	723.2103	499.1444/175.0398	1-sinapoyl-2-feruloyl-gentibiose	21.13 ± 1.31 ^b^	93.64 ± 7.32 ^d^	104.85 ± 3.16 ^e^	3.94 ± 0.62 ^a^	122.49 ± 3.25 ^f^	73.89 ± 1.98 ^c^
8.81	1139.2924	977.4442/285.1507	Kempferol-3-sinapoylsophorotrioside-7-glucoside	3.90 ± 0.24 ^a^	20.55 ± 1.02 ^e^	19.85 ± 1.08 ^d^	14.22 ± 0.52 ^c^	11.99 ± 1.12 ^b^	10.20 ± 0.52 ^b^
8.93	693.2132	499.1516/259.5630	1,2-diferuoyl-diglucoside	1.19 ± 0.14 ^c^	0.93 ± 0.24 ^c^	0.80 ± 0.08 ^b^	9.29 ± 0.32 ^d^	0.39 ± 0.36 ^a^	0.87 ± 0.26 ^b^
9.21	959.2661	735.3117/511.1374	Trisinapoyl-gentionbiose	2.72 ± 0.04 ^a^	62.78 ± 2.47 ^c^	62.23 ± 1.74 ^c^	85.71 ± 3.52	60.22 ± 0.76 ^b^	52.04 ± 1.14 ^d^
9.49	929.2539	723.2125	Feruloyl-disinapoyl-gentionbiose	1.99 ± 0.27 ^a^	18.96 ± 1.12 ^e^	16.63 ± 1.54 ^d^	5.85 ± 0.24 ^b^	22.22 ± 0.24 ^f^	11.90 ± 0.76 ^c^
9.81	1109.3099	947.0086/285.0297	Kempferol-3-feruloylsophorotrioside	5.32 ± 0.67 ^b^	10.00 ± 0.64 ^c^	9.86 ± 0.68 ^c^	2.61 ± 0.12 ^a^	9.77 ± 0.56 ^c^	6.86 ± 1.08 ^a^
10.07	929.2347	705.1979	Feruloyl-disinapoyl-gentionbiose	1.11 ± 0.17 ^b^	1.61 ± 0.18 ^b^	2.06 ± 0.16 ^c^	1.66 ± 0.32 ^b^	1.16 ± 0.06 ^b^	0.52 ± 0.36 ^a^
10.56	489.2639		Kaempferol 3-*O*-(6″-acetyl-hexoside)	9.01 ± 0.63 ^f^	2.11 ± 0.14 ^b^	1.66 ± 0.24 ^a^	6.90 ± 0.68 ^e^	4.28 ± 0.23 ^d^	3.62 ± 0.09 ^c^
10.73	959.2738	735.1979/511.2109	Trisinapoyl-gentionbiose	446.37 ± 0.42 ^e^	3.69 ± 0.08 ^b^	2.37 ± 0.35 ^a^	15.50 ± 0.54 ^d^	5.13 ± 0.42 ^c^	3.28 ± 0.11 ^b^
10.85	625.4776	/463.2062/301.0021	Quercetin-3,4′-*O*-di-beta-glucoside	0.81 ± 0.06 ^a^	2.43 ± 0.12 ^b^	2.86 ± 0.42 ^b^	166.88 ± 4.54 ^c^	2.21 ± 0.09 ^b^	2.44 ± 0.25 ^b^
			**Total [mg/100g]**	**1107.87 ± 33.79**	**466.92 ± 16.54**	**427.82 ± 13.12**	**739.64 ± 24.98**	**446.95 ± 11.86**	**371.85 ± 9.46**

Abbreviations: Rt—retention time; [M−H]^−^ (*m/z*)—masses of identified compounds; MS/MS fragments (*m/z*)—masses of identified fragments. Values (mean of three replications) ± standard deviation followed by different letters (a–f) are different (*p* ≤ 0.05) according to Duncan’s test. B—broccoli; B CD50 and B CD70—broccoli dried by convective drying at 50 and 70 °C, respectively; BB—broccoli after impregnation with beetroot juice; and BB CD50 and BB CD70—broccoli after impregnation with beetroot juice and dried by convective drying at 50 and 70 °C, respectively. ^a^ Experimental data. ^b^ Identification confirmed by commercial standards.

**Table 2:** Glucosinolates identified in broccoli (B) and broccoli impregnated in beetroot juice (BB) dried by convective drying at 50 °C (CD50) and 70 °C.

Rt (min)	[M−H]^−^	MS/MS	Compound
	(*m/z*)	Fragments (*m/z*)	
2.60	341.0860		Vulgaxanthin II
2.63	356.0997		Cyclodopa glucoside
2.93	385.1063		N-Formylcyclodopa glucoside
5.29	551.1737	389.1350	Betanin
9.15	535.1428		Mono-decarboxy betanin

Abbreviations: Rt—retention time; [M−H]^−^ (*m/z*)—masses of identified compounds; MS/MS fragments (*m/z*)—masses of identified fragments.

**Table 3:** Betalains identified in broccoli (B) and broccoli impregnated in beetroot juice (BB) dried by convective drying at 50 °C (CD50) and 70 °C.

Rt (min)	[M−H]^−^	MS/MS	Compound
	(*m/z*)	Fragments (*m/z*)	
0.90	195.0461	177.0476/129.0088	Gluconic acid
1.87	436.0401	372.0553/178.0165	Glucoraphanin
3.19	447.0482	259.0295/134.0551	Glucobrassicin
3.76	447.0535	254.1377/139.0027	Indolymethyl glucosinolate
4.66	422.0568	358.1490/259.0676	Glucoiberin
4.97	477.0605	284.0360/154.0144	Neoglucobrassicin
6.16	477.0588	235.9673	Methoxyglucobrassicin 1
6.32	477.0568	235.9234	Methoxyglucobrassicin 2

Abbreviations: Rt—retention time; [M−H]^−^ (*m/z*)—masses of identified compounds; MS/MS fragments (*m/z*)—masses of identified fragments.

**Table 4:** Antioxidant capacity (µMol Trolox/100 g of dry matter) and total phenolic content of broccoli (B) and broccoli impregnated in beetroot juice (BB) dried by convective drying at 50 °C (CD50) and 70 °C.

Method	ABTS	DPPH	FRAP	TPC
B	58.98 ± 2.28 ^f^	88.60 ± 3.13 ^e^	41.60 ± 2.22 ^e^	9.82 ± 0.58 ^d^
B CD50	19.07 ± 0.24 ^b^	21.78 ± 0.72 ^b^	15.50 ± 1.08 ^b^	2.96 ± 0.38 ^a^
B CD70	29.55 ± 0.74 ^d^	22.39 ± 0.38 ^b^	22.92 ± 1.73 ^d^	4.81 ± 0.46 ^c^
BB	44.79 ± 2.42 ^e^	66.03 ± 2.62 ^d^	47.79 ± 3.12 ^f^	13.06 ± 0.97 ^e^
BB CD50	16.97 ± 0.76 ^a^	16.57 ± 0.56 ^a^	13.20 ± 0.62 ^a^	2.63 ± 0.14 ^a^
BB CD70	24.07 ± 1.56 ^c^	24.34 ± 1.98 ^c^	19.72 ± 1.04 ^c^	3.50 ± 0.32 ^b^

Values (mean of three replications) ± standard deviation followed by different letters (a–f) are different (*p* ≤ 0.05) according to Duncan’s test. Abbreviations: ABTS—2,2′-azino-bis(3-ethylbenzothiazoline-6-sulfonic acid); DPPH—2,2-diphenyl-1-(2,4,6-trinitrophenyl)hydrazyl; FRAP—ferric reducing antioxidant power assay; B—broccoli; B CD50 and B CD70—broccoli dried by convective drying at 50 and 70 °C, respectively; BB—broccoli after impregnation with beetroot juice; BB CD50 and BB CD70—broccoli after impregnation with beetroot juice and dried by convective drying at 50 and 70 °C, respectively.

**Table 5:** Weight gain (WG), Brix degrees (ºBx), and dry mass (DM).

Material	WG (%)	°Bx	DM (%)
B	-	-	12.87 ± 0.57
BB	14.88 ± 0.43	10.3 ± 0.2	11.95 ± 0.43

B—broccoli; BB—broccoli after impregnation with beetroot juice.

**Table 6:** Parameter values a, b, k, R^2^, RMSE, χ^2^, and V_e_ of the function describe the drying kinetics for courgette samples dried by convection at different temperatures.

Drying Method	Material	Model Parameters			Statistical Parameters				Drying Time [min]
		k	a	b	RMSE	V_e_ [%]	R^2^	χ^2^	
Logistical Model									
CD50	B	0.0167	1962.7203	1870.3274	0.0165	5.1	0.9972	0.0003	290
	BB	0.0110	1589.9455	1488.6300	0.0990	5.6	0.9959	0.0004	350
CD70	B	0.0388	2.4187	3.3763	0.0072	2.1	0.9995	0.0001	150
	BB	0.0359	2.1818	3.1279	0.0093	2.9	0.9992	0.0001	170
Logarithmic Model									
CD50	B	0.0163	0.9270	0.0041	0.0113	3.8	0.9983	0.0001	290
	BB	0.0110	0.9320	0.0000	0.0989	5.6	0.9959	0.0004	350
CD70	B	0.0330	1.0199	0.0000	0.0134	4.6	0.9982	0.0002	150
	BB	0.0300	1.0149	0.0000	0.0153	5.5	0.9976	0.0003	170
Henderdon and Pabis Model									
CD50	B	0.0167	0.9525	-	0.01652	5.1	0.9972	0.0003	290
	BB	0.0110	0.9322	-	0.0199	5.6	0.9959	0.0004	350
CD70	B	0.0326	1.0097	-	0.0135	4.0	0.9986	0.0002	150
	BB	0.0297	1.0078	-	0.0150	4.8	0.9982	0.0003	170
Newton Model									
CD50	B	0.0179	-	-	0.0235	7.2	0.9972	0.0006	290
	BB	0.0121	-	-	0.0331	9.3	0.9941	0.0012	350
CD70	B	0.0322	-	-	0.0139	4.2	0.9988	0.0002	150
	BB	0.0294	-	-	0.0153	4.8	0.9984	0.0003	170
Page Model									
CD50	B	0.0297	0.8734	-	0.0075	2.3	0.9995	0.0001	290
	BB	0.0224	0.8620	-	0.0203	5.7	0.9961	0.0004	350
CD70	B	0.0276	1.0338	-	0.0071	2.2	0.9995	0.0001	150
	BB	0.0236	1.0624	-	0.0119	3.8	0.9987	0.0002	170

B—broccoli; BB—broccoli after impregnation with beetroot juice; CD50—dried by convective drying at 50 °C; and CD70—dried by convective drying at 70 °C.

**Table 7:** Water activity (AW) and density (ρb).

Method	Water Activity [-]	Density [kg/m^3^]
B	0.987 ± 0.007 ^e^	240.94 ± 5.05 ^d^
B CD50	0.469 ± 0.008 ^c^	111.34 ± 4.00 ^b^
B CD70	0.436± 0.001 ^a^	84.16 ± 1.73 ^a^
BB	0.382 ± 0.008 ^e^	288.90 ± 5.98 ^d^
BB CD50	0.354 ± 0.010 ^d^	116.16 ± 2.95 ^b^
BB CD70	0.459 ± 0.005 ^b^	93.75 ± 2.87 ^c^

Values (mean of three replications) ± standard deviation followed by different letters (a–e) are different (*p* ≤ 0.05) according to Duncan’s test. B—broccoli; B CD50 and B CD70—broccoli dried by convective drying at 50 and 70 °C, respectively; BB—broccoli after impregnation with beetroot juice; and BB CD50 and BB CD70—broccoli after impregnation with beetroot juice and dried by convective drying at 50 and 70 °C, respectively.

**Table 8:** Color parameters of raw and dried broccoli: L*—lightness; a* for (+) redness/(−) greenness and b* for (+) yellowing; BI—browning index; C*—saturation; and ΔE—total color of vegetables.

Color Parameters	B	B CD50	B CD70	BB	BB CD50	BB CD70
L	32.54 ± 1.09 ^e^	18.35 ± 0.59 ^c^	15.75 ± 0.91 ^b^	23.86 ± 0.56 ^d^	16.74 ± 0.40 ^b^	14.62 ± 1.02 ^a^
A	−5.12 ± 0.61 ^a^	−1.90 ± 0.16 ^b^	−0.66 ± 1.12 ^b^	1.35 ± 0.04 ^c^	2.85 ± 0.53 ^d^	3.40 ± 0.26 ^e^
B	6.04 ± 0.76 ^c^	10.24 ± 0.38 ^e^	7.95 ± 0.33 ^d^	1.36 ± 0.11 ^a^	7.45 ± 0.55 ^d^	5.56 ± 0.71 ^b^
C*	5.60 ± 0.78 ^b^	10.15 ± 0.38 ^e^	7.91 ± 0.34 ^d^	1.79 ± 0.08 ^a^	7.64 ± 0.54 ^d^	5.86 ± 0.68 ^b^
BI	1.99 ± 2.09 ^a^	62.31 ± 3.2 ^c^	61.85 ± 10.59 ^c^	12.03 ± 0.69 ^b^	80.45 ± 6.17 ^e^	77.62 ± 9.87 ^d^
∆E	-	15.15 ^b^	17.48 ^c^	11.80 ^a^	17.75 ^c^	19.85 ^d^

Values (mean of three replications) ± standard deviations followed by different letters (a–e) are different (*p* ≤ 0.05) according to Duncan’s test. B—broccoli; B CD50 and B CD70—broccoli dried by convective drying at 50 and 70 °C, respectively; BB—broccoli after impregnation with beetroot juice; and BB CD50 and BB CD70—broccoli after impregnation with beetroot juice and dried by convective drying at 50 and 70 °C, respectively.

**Table 9:** Material codes for raw and dried broccoli.

Code	Material	Type of Drying
B	Broccoli	-
B CD50	Broccoli	Convective drying at 50 °C
B CD70	Broccoli	Convective drying at 70 °C
BB	Broccoli after impregnation with beetroot juice	-
BB CD50	Broccoli after impregnation with beetroot juice	Convective drying at 50 °C
BB CD70	Broccoli after impregnation with beetroot juice	Convective drying at 70 °C

**Table 10:** Mathematical models.

Number	Model Name	Equation
1	Page Model [61]	$MR=exp(-k\cdot \tau^{a})$
2	Henderdon and Pabis Model [62]	MR=a·exp(−kτ)
3	Newton Model [63]	MR=exp(−kτ)
4	Logarithmic Model [53]	$MR=a\cdot \exp\left(-k\tau \right)+b\cdot exp(-k_{ja}\cdot \tau )$
5	Logistic Model [64]	$MR=\frac{b}{\left(1+a\cdot \exp\left(k\cdot \tau \right)\right)}$

k—drying coefficient [min^−1^]; a and b—coefficients of the equations; n—exponent; τ—time [min]; and MR—moisture ratio.

## Data Availability

Data are contained within the article.
